# Epidemiology of measles cases, vaccine effectiveness, and performance towards measles elimination in The Gambia

**DOI:** 10.1371/journal.pone.0258961

**Published:** 2021-10-21

**Authors:** Alieu Sowe, Mbye Njie, Dawda Sowe, Sidat Fofana, Lamin Ceesay, Yaya Camara, Brook Tesfaye, Samba Bah, Alieu K. Bah, Abdoulie K. Baldeh, Bakary D. Dampha, Samba N. Baldeh, Alagie Touray

**Affiliations:** 1 WHO Country Office, Nairobi, Kenya; 2 Expanded Program on Immunisation, Ministry of Health, Banjul, The Gambia; 3 World Bank Country Office, Cape Point, Bakau, The Gambia; 4 School of Public Health and Community Medicine, Institute of Medicine, University of Gothenburg, Gothenburg, Sweden; 5 Department of Global Public Health, Karolinska Institutet, Solna, Sweden; 6 WHO Country Office, Juba, South Sudan; Babol University of Medical Science, ISLAMIC REPUBLIC OF IRAN

## Abstract

**Introduction:**

In 2011, member states of the World Health Organization (WHO) Africa Regional Office (AFRO) resolved to eliminate Measles by 2020. Our study aims to assess The Gambia’s progress towards the set AFRO measles elimination target and highlight surveillance and immunisation gaps to better inform future measles prevention strategies.

**Material and methods:**

A retrospective review of measles surveillance data for the period 2011–2019, was extracted from The Gambia case-based measles surveillance database. WHO—UNICEF national coverage estimates were used for estimating national level MCV coverage. Measles post campaign coverage survey coverage estimates were used to estimate national measles campaign coverage.

**Results:**

One hundred and twenty-five of the 863 reported suspected cases were laboratory confirmed as measles cases. More than half (53.6%) of the confirmed cases have unknown vaccination status, 24% of cases were vaccinated, 52.8% of cases occurred among males, and 72.8% cases were among urban residents. The incidence of measles cases per million population was lowest (0) in 2011–2012 and highest in 2015 and 2016 (31 and 23 respectively). The indicator for surveillance sensitivity was met in all years except in 2016 and 2019. Children aged 5–9 years (Incidence Rate Ratio—IRR = 0.6) and residents of Central River region (IRR = 0.21) had lower measles risk whilst unvaccinated (Adjusted IRR = 5.95) and those with unknown vaccination status (IRR 2.21) had higher measles risk. Vaccine effectiveness was 89.5%.

**Conclusion:**

The Gambia’s quest to attain measles elimination status by 2020 has registered significant success but it is unlikely that all target indicators will be met. Vaccination has been very effective in preventing cases. There is variation in measles risk by health region, and it will be important to take it into account when designing prevention and control strategies. The quality of case investigations should be improved to enhance the quality of surveillance for decision making.

## Introduction

Measles is a highly infectious vaccine-preventable viral disease that accounted for at least 2 million annual global deaths before the introduction of the measles vaccine in 1963 [1]. It spreads rapidly and has a basic reproductive number (R0) of approximately 12–18 [2]. In 2001, the WHO set a goal to reduce global measles mortality and urged member states to fully implement the strategies for measles mortality reduction stated in WHO-UNICEF strategic plan 2001–2005 [3]. These strategies are: 1) providing the first dose of measles vaccine to successive cohorts of infants; 2) ensuring that all children have a second opportunity for measles vaccination; 3) enhancing measles surveillance with integration of epidemiological and laboratory information and; 4) improving the management of every measles case [4]. The Global Vaccine Action Plan (GVAP) 2011–2020, building on previous initiatives against measles, ambitiously targeted measles elimination in at least five WHO regions by the end of 2020 [5]. The “Framework for verifying elimination of measles and rubella” [6] and the “Guidance for evaluating progress towards elimination of measles and rubella” [7] provide guidance in measles and rubella elimination. By 2018, global measles deaths decreased to 142,000 from 536,000 in 2000, indicating a reduction of 73% [1].

In 2011, member states of the WHO Africa Regional Office (AFRO) resolved to eliminate measles by 2020 and made the following targets as indicators of achieving elimination status by countries in the AFRO region: 1) a national measles incidence of less than 1 case per million population; 2) at least 95% measles immunization coverage at national and district levels; 3) at least 95% coverage in all measles supplementary immunisation activities (SIAs); 4) one or more suspected measles cases investigated in at least 80% of districts per year and; 5) a national non-measles febrile rash illness rate (NMFRI) of at least 2 per 100 000 population [8]. A report published in the *Morbidity and Mortality Weekly Report* describing global progress towards measles elimination from 2000 to 2018 acknowledged the significant progress made but stressed the need for improving routine immunisation and surveillance amongst others for regional elimination targets to be met [9]. Country level studies conducted in several countries have also highlighted gaps that need to be addressed to achieve and sustain measles elimination [10–18]. The AFRO region has the highest incidence of measles [9]. Elimination achievement and sustenance require sufficiently high measles vaccination coverage in the presence of a robust surveillance system in each country.

A strong surveillance system is required to detect and respond to measles outbreaks. The declaration of a measles outbreak could be context specific dependent on incidence. Response strategies to measles outbreaks may include a) putting in place mechanisms to reach vulnerable groups in terms of geographical location, b) identifying weaknesses in immunization and surveillance systems, c) enhancing social mobilization and community participation and, d) understanding the distribution of measles cases over time [19]. The minimum measles surveillance standards set by AFRO for countries in the region are an important proxy in monitoring performance towards elimination. Country level achievement and sustenance of measles elimination are essential for achieving and maintaining regional level measles elimination. The WHO has established regional verification committees (RVCs) in each of the six WHO regions and recommended that each country establishes a national verification committee (NVC) to annually review performance towards measles elimination through a standardised but adaptable process [7]. The Gambia is yet to have a NVC. Cognizant of the challenges some countries would have in providing all the evidence needed for verification, the elimination evaluation guiding document gave RVCs leeway to use complementary evidence at their discretion [7].

One of the major threats to vaccine utilization may be linked to vaccine hesitancy which is emerging in many developing countries in recent times [20]. Generally, Gambian women are receptive to vaccination services [21]. The first dose of the measles vaccine was introduced in the early 1980s whilst the second dose was introduced in 2012 [22]. In The Gambia and some countries, the first dose of measles vaccine is administered subcutaneously as a 0.5ml dose of antigen given at 9 months of age, and children receive their second dose of measles at 18 months of age. Per WHO—UNICEF national coverage estimates for the first dose of measles containing vaccine (MCV1), The Gambia is one of the best performing countries in the AFRO region [22]. The country’s national MCV1 coverage has been between 90% and 97% from 2011 to 218 and declined to 85% in 2019. MCV2 coverage was 56% in 2012, the year it was introduced, and 61% in 2019. The country conducted 2 SIA from 2011 to 2019 targeting children 9 months to 14 years. The coverages of those SIAs were 96% and 97% respectively. Despite relatively high coverage, there have been sporadic measles outbreaks over time [23]. Therefore, identifying and addressing coverage gaps in countries is critical to accelerating and maintaining measles elimination.

When Masresha et al. (2018) included The Gambia in their 2012–2016 assessment of the elimination status of measles in 11 African countries with high routine MCV1 coverage [17], they concluded that none of the countries assessed were ready for the verification of their measles elimination status [17]. Wariri et al. (2021) developed a scorecard which they used to track the progress of 15 West African (ECOWAS) countries towards measles control and elimination milestones between 2001 and 2019 [24]. They found that although 3 countries including The Gambia have made substantial achievements to measles, the ECOWAS block is not track to achieving measles elimination in 2020 [24]. Since their aim was to mainly assess progress towards measles elimination, Masresha et al. (2018) and Wariri et al. (2021) did not include further analysis such as the epidemiology of cases, risk factors, and vaccine effectiveness, which would also be useful in informing measles control and elimination strategies at country level [17, 24]. In addition to that, the occurrence of sporadic measles outbreaks in parts of the country over time, points to immunity gaps and would require further investigation to effectively address such gaps. Identification of MCV vaccination immunity gaps requires a thorough assessment of MCV coverage over time, vaccine effectiveness, cold chain issues, human resource for EPI services, social mobilization, and epidemiological investigation of reported measles cases including measles risk factors amongst others. It is against this background that this study aims to assess The Gambia’s progress towards the set AFRO measles elimination target and highlight surveillance and immunisation performance to better inform future measles elimination strategies.

## Materials and methods

### Setting

The Gambia is West African country with a population of about 2.4 million people. It is bothered to the west by the Atlantic Ocean and to the north, south, and east by Senegal. The country has a human development index of 0.466 [25]. The Gambia Expanded Program on Immunisation (EPI) was started in 1979 after an outbreak of Yellow Fever. The country has seven health regions which are also considered as reporting districts for immunisation and surveillance purposes. In each of the seven health regions, is a Regional Operations Officer for the national EPI who is responsible for EPI program activities in that region.

### Study design and data sources

A retrospective review of measles surveillance data for the period 2011–2019, was extracted from The Gambia case-based measles surveillance database. WHO—UNICEF national coverage estimates were used for estimating national level MCV coverage [22]. Measles post campaign coverage survey (PCCS) coverage estimates were used to estimate national measles–rubella vaccination campaign conducted in 2016 [26].

### Measles case definitions (outcome variable)

A suspected measles case is defined in The Gambia as any person with fever and generalized maculo-papular rash plus one of the following: cough or coryza (runny nose) or conjunctivitis (red eyes) **or** any person in whom a clinician suspects measles. A laboratory confirmed measles case is a suspected case of measles that has been confirmed positive by testing in a proficient laboratory, and vaccine-associated illness has been ruled out. All measles cases in the analysis were laboratory confirmed cases.

### Selected measles elimination indicators

To track the country’s status towards the elimination goal, we estimated the five core indicators over the study period stated in the AFRO measles elimination resolution. The five indicators and how each was calculated is presented below.

#### 1) National measles incidence rate per million population

For each of the years in the study period, the total measles cases for the year as per the database were divided by the estimated national population for that year then multiplied by 1,000,000.

#### 2) National measles routine immunization coverage

National MCV1 and MCV2 coverages over the years were extracted from WHO-UNICEF estimates national vaccination coverages [22].

#### 3) Measles coverage in SIAs conducted during the study period

We used the 2011 campaign report and 2016 measles PCCS coverage estimates.

#### 4) Proportion of districts investigating at least 1 measles case per year

For this indicator, the number of districts which investigated at least one suspected measles case was divided by the total number of districts expected to report suspected measles cases in that year. The results were converted to percentages by multiplying them by 100. There were six reporting districts in 2011–2012 and seven reporting districts 2013–2019 because Western Region 1 and Western Region 2 were one region (Western Region) before 2013.

#### 5) National non-measles febrile rash illness (NMFRI) rate

This was calculated by taking the total number of discarded (non-measles) cases reported per year and dividing it by the estimated national population in that year then multiplying the result by 100,000 in line with the standard way of calculating the indicator.

### Epidemiologic variables (including risk factors)

The epidemiology of cases was described using sex, age, vaccination status, residence type, and health region. All variables were categorized. Sex was categorised as male and female; age was categorised into three–under 5 years, 5–9 years, and 10 years & above. Vaccination status was indicated by vaccinated, unvaccinated, and unknown; residence as urban and rural; and health regions (reporting districts) were listed as Western Region 1, Western region 2, North Bank West, North Bank East, Lower River, Central River, and Upper River. Before 2013, Western Region 1 and Western Region 2 were administered as one reporting district called Western Region.

### Vaccine effectiveness

Vaccine effectiveness was calculated using the formula below [27]. A total of 863 suspected measles cases were reported during the period reviewed. Four hundred and ten (410) of the reported cases have unknown vaccination status and 49 of them were less than 9 months of age. All cases with unknown vaccination status or below 9 months (total 459) were excluded from this analysis. A case was considered vaccinated if he/she received at least one dose of MCV either by card or recall including SIA doses.


$$VE=\frac{ARU-ARV}{ARU}\times 100$$

Where:

VE = Vaccine Effectiveness (in percentage), ARU = Attack Rate in the Unvaccinated, and ARV = Attack Rate in the Vaccinated. The ARU was calculated as the incidence of measles in unvaccinated and ARV was calculated as the measles incidence in the vaccinated.

### Approach to analysis

#### Main analysis

Selected measles elimination status indicators, epidemiology of cases, and vaccine effectiveness were descriptively analysed using rates or proportions. Risk factors were identified through Poisson regression. The motivation for choosing a Poisson model is to enable the estimation of rate ratios since the outcome is relatively common (>10%). Therefore, a logistic model would tend to overestimate the strengthen of associations when used. Exposure variables with P values ≤ .20 in bivariable analysis were included in the regression models. Two regression models were used. The first one is a crude model in which the outcome variable (in this case measles IgM positivity status indicated as a “1” and a “0” representing a “Yes” and a “No”) was regressed on each of the exposure variables. The second model is a fully adjusted model whereby the outcome variable was regressed on all the exposure variables at once. Incidence rate ratios and their 95% confidence intervals were reported. Data management and analysis were done using Stata 16.1 [28].

#### Additional analysis

Seven observations had missing age. Three of them had measles IgM positive results. All the seven observations were recoded into a category called “missing” and included in the regression models, then it was dropped, and the regressions were repeated. The finding was that dropping them had no major effect on the regression results. And therefore, they were dropped. The possibility of multicollinearity among independent variables was assessed by estimating variance inflation factors (VIFs). As a rule of thumb VIFs below 5 were considered non problematic. The highest VIF was 1.73, suggesting that multicollinearity might not be a problem in the adjusted model. The model goodness of fit P—value for the adjusted model was 1 (good fit should be > 0.05 –the higher the P value, the better the model) indicating that the adjusted model reasonably fits the data well.

### Ethical considerations

This study used routine national surveillance data. Therefore, no ethical approval was required. The subset of the data extracted for analysis was fully anonymized before it was accessed for analysis.

## Results

### Description of measles cases

A total of 863 suspected measles cases were reported across the 9 years analysed. Of these, 125 were confirmed measles cases. As can be seen in Table 1, most measles cases occurred in 2015 and 2016. By sex, 52.8% of cases were males. Cases tend to vary by age with the frequency of cases decreasing with age e.g., 46.4% among children below 5 years and 18.4% among those aged 10 years and above. Three cases representing 2.4% of the total measles cases had missing age. More than half (53.6%) of the cases have unknown vaccination status and 24% of the total cases were vaccinated. A little less than three quarters (72.8%) of all cases were among urban residents. Most cases were from Western Region 1 (44%) and Western Region 2 (34.4%) followed by Upper River region (11.2%). North Bank West and Central River regions accounted for the lowest frequency of cases—at 0.8% each.

**Table 1:** Description of laboratory confirmed measles cases in The Gambia 2011–2019.

Variable	2011 n (%)	2012 n (%)	2013 n (%)	2014 n (%)	2015 n (%)	2016 n (%)	2017 n (%)	2018 n (%)	2019 n (%)	Total n (%)
***Total cases (N)***	***0***	***0***	***0***	***1***	***61***	***57***	***1***	***4***	***1***	***125***
***Sex***										
Female	0 (0)	0 (0)	0 (0)	1 (100)	25 (41)	32 (56.1)	0 (0)	1 (25)	0 (0)	59 (47.2)
Male	0 (0)	0 (0)	0 (0)	0 (0)	36 (59)	25 (43.9)	1 (100)	3 (75)	1 (100)	66 (52.8)
***Age***										
Under 5 years	0 (0)	0 (0)	0 (0)	1 (100)	35 (57.4)	20 (35.1)	1 (100)	1 (25)	0 (0)	58 (46.4)
5–9 years	0 (0)	0 (0)	0 (0)	0 (0)	21 (34.4)	17 (29.8)	0 (0)	3 (75)	0 (0)	41 (32.8)
10 & + years	0 (0)	0 (0)	0 (0)	0 (0)	5 (8.2)	17 (29.8)	0 (0)	0 (0)	1 (100)	23 (18.4)
Missing	0 (0)	0 (0)	0 (0)	0 (0)	0 (0)	3 (5.3)	0 (0)	0 (0)	0 (0)	3 (2.4)
***Vaccination status***										
Unvaccinated	0 (0)	0 (0)	0 (0)	0 (0)	17 (27.9)	10 (17.5)	1 (100)	0 (0)	0 (0)	28 (22.4)
Vaccinated	0 (0)	0 (0)	0 (0)	0 (0)	17 (27.9)	12 (21.1)	0 (0)	1 (25)	0 (0)	30 (24)
Unknown	0 (0)	0 (0)	0 (0)	1 (100)	27 (44.3)	35 (61.4)	0 (0)	3 (75)	1 (100)	67 (53.6)
***Residence***										
Rural	0 (0)	0 (0)	0 (0)	1 (100)	12 (19.7)	17 (29.8)	0 (0)	4 (100)	0 (0)	34 (27.2)
Urban	0 (0)	0 (0)	0 (0)	0 (0)	49 (80.3)	40 (70.2)	1 (100)	0 (0)	1 (100)	91 (72.8)
***Health Region***										
Central River	0 (0)	0 (0)	0 (0)	0 (0)	0 (0)	1 (1.8)	0 (0)	0 (0)	0 (0)	1 (0.8)
Lower River	0 (0)	0 (0)	0 (0)	0 (0)	2 (3.3)	1 (1.8)	0 (0)	0 (0)	0 (0)	3 (2.4)
North Bank East	0 (0)	0 (0)	0 (0)	1 (100)	5 (8.2)	2 (3.5)	0 (0)	0 (0)	0 (0)	8 (6.4)
North Bank West	0 (0)	0 (0)	0 (0)	0 (0)	0 (0)	1 (1.8)	0 (0)	0 (0)	0 (0)	1 (0.8)
Upper River	0 (0)	0 (0)	0 (0)	0 (0)	2 (3.3)	8 (14)	0 (0)	4 (100)	0 (0)	14 (11.2)
Western 1	0 (0)	0 (0)	0 (0)	0 (0)	29 (47.5)	24 (42.1)	1 (100)	0 (0)	1 (100)	55 (44)
Western 2	0 (0)	0 (0)	0 (0)	0 (0)	23 (37.7)	20 (35.1)	0 (0)	0 (0)	0 (0)	43 (34.4)

### Selected measles elimination status indicators

Table 2 depicts The Gambia’s performance towards measles elimination during 2011–2019. The proportion of districts investigating at least 1 suspected measles case for which blood specimen was collected was the only indicator that was consistently met from 2011 to 2019. There was one SIA conducted during the period reviewed and national coverage was 97%, 2 percentage points above the minimum target. The incidence of measles cases per million population was lowest (0) in 2011–2012 and highest in 2015 and 2016 (about 31 and 23 respectively). Although MCV1 coverage achieved met the set target in 5 of the 9 years assessed, MCV2 coverage has been below the 95% target since its introduction in 2012 and has a median coverage of 69.5%. The indicator for surveillance sensitivity, the NMFRI rate, was met in all years except in 2016 and 2019.

**Table 2:** Selected measles elimination status indicators, The Gambia 2011–2019.

Measles elimination status indicator	Target	Achieved								
		2011	2012	2013	2014	2015	2016	2017	2018	2019
Measles incidence rate per million	Less than 1	0	0	0	0.52	30.74	22.91	0.47	1.82	0.44
Routine MCV^1^ 1 coverage	At least 95%	91%	95%	96%	96%	97%	97%	90%	91%	85%
Routine MCV 2 coverage	At least 95%	NA	56%	53%	73%	81%	79%	68%	71%	61%
MCV coverage during SIAs	At least 95%	96%	NA	NA	NA	NA	97%	NA	NA	NA
% of districts investigating 1 suspected case/year	At least 80%	83%	83%	86%	100%	86%	100%	100%	100%	100%
Non-measles febrile rash illness (NMFRI) rate	At least 2	6.22	5.02	7.27	5.05	5.19	1.32	2.69	2.73	1.94

### Vaccine effectiveness

Four hundred and four (404) of the 863 reported suspected cases have known vaccination status and were above 9 months of age and therefore eligible for inclusion in this analysis. The attack rates were 66.67% and 6.98% among the unvaccinated and vaccinated respectively leading to vaccine effectiveness rate of 89.5%.

### Measles risk factors

Table 3 shows exposure variables and their association with measles during the period studied. Children aged 5–9 years (Adjusted IRR = 0.6) and residents of Central River region (Adjusted IRR = 0.21) had lower risk of contracting measles than their respective reference categories. Upper River and Western Region 2 residents respectively have 2.52- and 1.8-folds higher measles risk than residents of Western Region 1. Those unvaccinated (Adjusted IRR = 5.95) and those with unknown vaccination status (Adjusted IRR 2.21) had much higher measles incidence than those vaccinated.

**Table 3:** Measles risk factors in The Gambia 2011–2019.

Variables	Crude^1^ IRR^2^ (P-value) (N = 856)	Adjusted^3^ IRR (P-value) (N = 856)
***Age***		
Under 5 years	Reference	Reference
5–9 years	0.63 (0.01)	0.62 (0.01)
10 & above years	0.88 (0.58)	0.78 (0.27)
***Vaccination status***		
Vaccinated	Reference	Reference
Unvaccinated	7.55 (<0.01)	5.74 (<0.01)
Unknown	2.2 (<0.01)	2.23 (<0.01)
***Residence***		
Rural	Reference	Reference
Urban	2.00 (<0.01)	1.43 (0.11)
***Health region***		
West Coast 1	Reference	Reference
Central River	0.14 (<0.01)	0.21 (0.23)
Lower River	0.09 (0.02)	0.14 (0.05)
North Bank East	1.01 (0.98)	1.61 (0.21)
North Bank West	0.21 (0.12)	0.38 (0.33)
Upper River	2.06 (0.01)	2.52 (<0.01)
West Coast 2	1.73 (<0.01)	1.80 (<0.01)

^1^ Variables were individually regressed on the measles IgM result variable.

^2^ IRR means Incidence Rate Ratio.

^3^ Variables were fully controlled for each other in the adjusted model.

## Discussion

The WHO AFRO region, including The Gambia, targeted year 2020 as the year to eliminate measles. The achievement of five key measles elimination indicators is the required for measles elimination to be declared at country level. Each country in the AFRO region must achieve and sustain the targets for the region to be considered to have achieved measles elimination. We tracked measles elimination status in The Gambia over the last 9 years from 2011, by epidemiologically describing laboratory confirmed measles cases, evaluating vaccine effectiveness, and identifying risk factors. Our findings highlight that significant achievements have been registered in The Gambia towards measles elimination including high immunization coverage. Nonetheless, the study also illuminated gaps that require attention to reinforce gains made over the past decades in order to accelerate measles elimination.

Over the nine years evaluated, there is no single year in which all the five indicators recommended by AFRO were met [8]. The finding that each of the reporting districts was able to report at least 1 case per year during the period reviewed highlights that the country has a sensitive surveillance system which is very positive. Although the incidence of measles cases peaked in 2015 and has remained significantly lower since then, the maintenance of the recommended threshold remains a challenge. Reasons given by national staff for the spike in cases in 2015 include a delay in implementing a planned SIA and low MCV 2 coverage. Most of the cases were recorded among foreigners whose vaccination statuses were mostly either unvaccinated or unknown. The decreasing trend of MCV1 and MCV2 coverage rates in 2019 compounded by the intermittent achievement of the surveillance sensitivity target in half of the years after 2015 creates an environment suggesting that The Gambia may not be able to achieve measles elimination by the targeted year.

With regards to the epidemiology of measles cases, interesting and sometimes expected patterns have been observed. Majority of absolute cases occurred among males but proportions of cases by sex are not statistically different. Studies conducted in Nigeria, Ethiopia, Kenya, South Korea, and Iraq also reported higher frequencies of measles cases among males [11–13, 16, 18]. The presence of few confirmed measles cases with missing age and a majority of confirmed cases having unknown vaccination status despite over three quarters (79%) of all measles occurring among children less than ten years points to data quality related issues. Vaccination card retention is over 80% in The Gambia [29–32] suggesting that missingness in variables or having unknown vaccination status could be related to the timeliness of investigations and complete filling of case investigation forms. Kisangau et al.’s (2018) study tracking progress towards measles elimination in Kenya made findings similar to ours in that most measles cases had unknown vaccination status [16]. Unknown vaccination status of cases negatively affects surveillance data quality and consequently decision making [33]. Unsurprisingly, individuals with unknown vaccination status and the unvaccinated persons have higher risk for measles infection than the vaccinated, even though study participants with unknown vaccination status appear to have a lower risk than those unvaccinated. It has been shown that measles cases are usually more common among those with unknown or missing vaccination status, after those classified as the unvaccinated [34, 35]. This suggests that the group with unknown vaccination status may be heterogeneous with majority of them being the unvaccinated than vaccinated.

Our study has shown variation in measles infection risk by geographic groups. It is quite interesting that urban residence was associated with higher likelihood of measles infection than rural residence but only in the crude model. The association disappeared when the variables were controlled for each other in the adjusted model. Regional, district, county or state variations in the distribution of measles cases is not uncommon [11, 13, 16, 18]. Higher vaccination coverage is usually associated with lower incidence of measles and it is historically known that vaccination coverage is higher in rural than urban areas in The Gambia [36]. Western Region 1 and Western Region 2 densely populated and account for over 60% of national population and are more urban than the rest of health regions with Western Region 1 being considered fully urban by surveys. Taking into account that the vast majority of confirmed cases occurred in those two regions, it is reasonable that health region explained (confounded) the association between residence and measles infection in this case.

Our study showed that MCV vaccine effectiveness was very high during the period reviewed (considering an MCV vaccine effectiveness threshold of 85% when given at 9 months [19]) unlike what was found in Kerela, India, where low vaccine effectiveness was reported [37]. An earlier study assessing measles mortality and vaccine efficacy in The Gambia by Hull et al. (1983) also reported as high as 89% vaccine efficacy among children vaccinated at 9 months or later [38]. Generally, measles vaccine effectiveness is improves with number of doses e.g. two doses are more effective in preventing measles than 1 dose. Therefore, SIAs also play an important role in boosting population immunity induced through routine immunization.

### Limitations

Since the data collected is over nine years, the children in 5–9 years age cohort is not exactly the same across the 9 years reviewed. Therefore, it is important to keep in mind when interpreting results that children grouped 5–9 years is actually an aggregate of children who were in that age group in each of the years and not necessarily the total number of children aged 5–9 years over the study period. This caveat is worth noting to avoid misinterpreting results by assuming that children born five to nine years before the beginning of the study period (2011) were more vulnerable to measles. Another caveat is that the proportions of cases with missing and unknown vaccination status are calculated among measles IgM positive cases. Therefore, the absolute number of observations with missing age or unknown vaccination status will be higher if they were calculated across all the observations in the database.

## Policy and research implications

The findings of this study have presented a clear description of measles elimination status in The Gambia and provided insights that can be used to guide stakeholders to strengthen the planning and implementation of measles prevention strategies. The study has shown that a one size fits all approach will not work well to address in meeting measles elimination status indicators in The Gambia. An integrated and context specific approach is needed among programs and all stakeholders to address the challenges. Not much country focused (The Gambia) research has been done in this area. It is therefore envisaged that this study will trigger interest and more research on vaccine-preventable disease surveillance in The Gambia with a view of providing information that actors can use to accelerate the prevention and control of vaccine preventable diseases. There is a need to investigate the declining trend in MCV coverage. The country needs to establish a national verification committee to monitor measles elimination status annually as recommended in the measles elimination evaluation guiding document [7].

## Conclusion

The Gambia’s quest to attain measles elimination status by 2020 has registered significant success but has a daunting challenge. The country’s performance towards the set elimination status indicators has been fluctuating and it is unlikely that all indicator targets will be met by 2020. There is variation in measles risk. Younger age groups and being resident in more urban areas were positively associated with measles infection. Vaccine effectiveness was shown to be very good. However, there is a need to improve on data quality to improve the quality of evidence that may be generated from surveillance date to guide future action.
